# NOVEL INVENTION OF SPORE INDUCTION IN A SISTER SPECIES TO GROUP 4 DICTYOSTELIA

**DOI:** 10.12688/openreseurope.18365.2

**Published:** 2024-12-05

**Authors:** Pauline Schaap

**Affiliations:** 1 School of Life Sciences, University of Dundee School of Life Sciences, Dundee, Scotland, DD15EH, UK

**Keywords:** developmental network innovation, Polysphondylium violaceum, Dictyostelium discoideum, induction of sporulation, chemoattractant, cyclic AMP

## Abstract

**Background:**

Dictyostelia are soil amoebas that aggregate to form fruiting bodies with spores and stalk cells in response to starvation. Where known, species across the dictyostelid phylogeny use secreted cAMP, detected by cAMP receptors (cARs) to induce the differentiation of spores and to organize fruiting body construction. However, recent deletion of the single *cAR* of *Polyspondylium violaceum (Pvio*) left both its fruiting bodies and spores intact.

**Methods:**

To investigate whether *Pvio* sporulation can occur in the absence of secreted cAMP and to explore alternative inducers in a bioassay *,* three prespore genes were identified and gene fusions of their promoters with the *LacZ* reporter gene were transformed into *Pvio* cells. After assessing the spatial expression pattern of the genes and the stage at which prespore gene expression initiated, the effect of cAMP and other *Dictyostelium discoideum* ( *Ddis*) signal molecules were tested on prespore gene expression *in vitro*.

**Results:**

*Pvio* genes *g4562 (psp1)*, *g2696 (psp2)* and *g2380 (psp3)* were identified as homologs of *Ddis* spore coat genes. They were first expressed around 4 h of starvation in aggregation centres and later in the posterior 4/5 ^th^ of emerging sorogens and the spore head of early fruiting bodies. Cells from dissociated 4 h aggregates and shaken in suspension for 6 h increased prespore- *LacZ* reporter activity 4-fold for *psp1* and 6-fold for *psp2,* but this increase was at least 5-fold higher when cells were plated on solid substratum for 6 h to develop normally. cAMP had no effect on prespore gene induction and neither had the *Pvio* chemoattractant glorin nor the *Ddis* chemoattractants and differentiation inducers folate, c-di-GMP, DIF-1, GABA, cGMP and 8Br-cAMP.

**Conclusions:**

The *Pvio* lineage uniquely evolved a novel genetic network for synthesis, detection and processing of the signal that triggers its main survival strategy.

## Plain language summary

The Dictyostelia are single-celled amoebas that eat soil bacteria. They aggregate to form a multicellular organism when food is depleted and construct a fruiting body with resilient spores, which become dispersed to new feeding grounds. Cyclic AMP (cAMP) plays a major role in this process as a secreted chemoattractant that brings the cells together and induces the expression of spore genes. cAMP also functions as an intracellular messenger for other signals that regulate the timely maturation of spores and stalk cells in the fruiting body.

The intracellular role of cAMP can be retraced to ancestors of the Dictyostelia, the solitary amoebas, which form less resilient cysts upon starvation. Here starvation and other stress conditions directly increase intracellular cAMP that then causes cells to encyst. All investigated Dictyostelia, including *Polyspondylium violaceum* that was studied here, use intracellular cAMP for spore and stalk cell maturation. However, *P. violaceum* is thus far the only species that can form fruiting bodies and spores without the receptors to detect cAMP. Does that mean that they also do not use secreted cAMP?

To answer that question a bioassay was developed to directly test effects of cAMP and other potential signal molecules on the expression of prespore genes. In the assay a low level of prespore gene expression was induced by an unknown signal that the cells themselves produced, but neither cAMP nor any of the six other signals that were tested had any effect on the expression of prespore genes. This means that *P. violaceum* evolved a whole set of novel genes to produce the unknown signal in a timely manner and to detect and process it. This is a major innovation in evolution and it will be of great interest to discover how this occurred and what the novel signal is.

## Introduction

Dictyostelia evolved multicellularity independently from animals, plants and fungi within the single-celled Amoebozoa. They feed on bacteria but unlike their amoebozoan relatives, which survive starvation and other stressors as individual dormant cysts, dictyostelid amoebas aggregate instead to form fruiting bodies with dormant spores and up to three supporting cell types. Molecular phylogenetics subdivides Dictyostelia in four major and some minor groups ( Schaap *et al.*, 2006; Schilde *et al.*, 2019). Group 4 contains the model organism *Dictyostelium discoideum* ( *Ddis*) and displays several traits that set it apart from the other groups. Group 4 species uniquely use cyclic AMP (cAMP), secreted in pulses, as chemoattractant for aggregation. They mostly form large solitary fruiting bodies with spores supported by stalk, basal disc and upper and lower cup cells and have an intermediate migrating slug stage in which future (pre) spore and supporting cells initiate differentiation in appropriate proportions required for fruiting bodies construction. Species in groups 1–3 generally form smaller clustered and/or branched structures with at most two cell types, spores and stalk cells. Here most cells differentiate as prespore cells and transdifferentiate into stalk cells at the fruiting body tip. Species in these groups often use the dipeptide glorin as chemoattractant. Many group 1, 2 and 3 species can also still encyst individually when stressed, but this alternative life cycle is lost in group 4 ( Romeralo *et al.*, 2013; Schilde *et al.*, 2014).

In addition to its role as chemoattractant, secreted cAMP acting on cAMP receptors (cARs) also induces the differentiation of prespore cells in group 4 ( Schaap & Van Driel, 1985; Wang *et al.*, 1988), while intracellular cAMP acting on cAMP dependent protein kinase (PKA) induces the maturation of spores and stalk cells ( Harwood *et al.*, 1992; Hopper *et al.*, 1993; Mann *et al.*, 1994). Evolutionary comparative studies showed that the roles of intracellular cAMP and PKA are conserved throughout Dictyostelia and are ancestrally derived from roles for cAMP and PKA as intermediates for stress-induced encystation in the solitary Amoebozoa ( Du *et al.*, 2014; Funamoto *et al.*, 2003; Kawabe *et al.*, 2015; Ritchie *et al.*, 2008). While secreted cAMP pulses did not mediate aggregation in groups 1–3, they did organise fruiting body morphogenesis in the group 3 species *D. minutum* and the group 2 species *Polysphondylium pallidum (Ppal)* ( Alvarez-Curto *et al.*, 2005; Schaap *et al.*, 1984). Secreted cAMP acting on cARs was also essential for induction of prespore differentiation in *P. pallidum* ( Kawabe *et al.*, 2009).

These observations suggested an evolutionary scenario in which the roles of cAMP in spore and stalk maturation, induction of prespore differentiation, organisation of morphogenesis and finally in group 4 aggregation were sequentially derived from the ancestral role of cAMP as intermediate for stress-induced encystation ( Schaap, 2016). Recent studies in *Polysphondylium violaceum (Pvio)*, which resides in a small sister clade to group 4, question the universality of this scenario. Unlike *Ppal,* where deletion of *car* genes disorganizes post-aggregative morphogenesis and prevents prespore and spore differentiation, deletion of the single *Pvio carA* gene leaves both its morphogenesis and spore differentiation intact ( Kawabe & Schaap, 2023).

While it remains possible that a second *car* remained unsequenced in its draft genome ( Narita *et al.*, 2020), lack of prespore markers made direct assessment of prespore gene induction by cAMP impossible. In this study, I identified relatives of three *Ddis* prespore genes in *Pvio*. *Pvio* cells transformed with gene fusions of the promoters of either gene and the *LacZ* reporter gene were used to study activation of the promoters by cAMP, glorin and signals known to promote or inhibit prespore differentiation in *Ddis*. The study indicated that the *Pvio* lineage invented a novel mechanism for prespore induction.

## Methods

### Cell culture

*Polysphondylium violaceum* strain QSvi11 ( Kalla *et al.*, 2011) was grown in association with *Klebsiella aerogenes* on 1/3 ^rd^ SM agar (13.9 g SM agar (catalogue number SMA0102 Formedium), 11 g agar (AGA03, Formedium), 1.27 g KH _2_PO _4_ (P5379, Sigma-Aldrich), 0.87 g K _2_HPO _4_.3H _2_O (P3786, Sigma-Aldrich) and 0.67 g MgSO _4_.7H _2_O (230391, Sigma-Aldrich) per litre H _2_O). Cells were harvested in PB (5 mM Na _2_HPO _4 _(71643, Sigma-Aldrich), 5 mM KH _2_PO _4_, pH 6.5), when about 1/3 ^rd^ of the bacterial lawn was cleared and developed on NN agar (1.5% agar in 8.8 mM KH _2_PO _4_ and 3.4 mM Na _2_HPO _4_) at 1.7 × 10 ^6^ cells/cm ^2^ until the desired developmental stage was reached.

### Promoter- *lacZ* constructs

To prepare promoter- *lacZ* fusions of the putative *Pvio* prespore genes g4562 ( *psp1*), g2696 ( *psp2*) and g2380 ( *psp3*), their respectively 2.1, 1.2 and 2.2 kb 5′intergenic regions were amplified from *Pvio QSvi11* genomic DNA using Phusion high-fidelity DNA polymerase (F530S, ThermoFisher Scientific) and the oligonucleotide primer pairs g4562pF/g4562pR, g2696pF/g2696pR or g2380pF/g2380pR ( Table 1) which harbour XbaI and BglII sites in the forward (F) and reverse (R) primers respectively. After XbaI/BglII digestion, the amplified products were cloned into XbaI/BglII digested vector pDdgal-17 ( Harwood & Drury, 1990), which fuses the start codon and usually the first few amino-acids of the inserted gene in frame with *LacZ*. The constructs were validated by DNA sequencing using primers Gal17F and Gal17R ( Table 1) and transformed into *Pvio QSvi11* as described by ( Narita *et al.*, 2020), except that initial overnight starvation was at 5×10 ^6^ cells/ml instead of 5×10 ^5^.

**Table 1.** Oligonucleotide primers.

Primer ID	Sequence	gene name
g4562pF	GGG **TCTAGA**GGGTAGGAGAGGTAACAAATG	*psp1*
g4562pR	GGG **AGATCT**TGACTTCATTTTATTCTTATTAATTTAATTC	
g2696pF	CCC **TCTAGA**CATATGATAGGTGTATGCAGATTTAAG	*psp2*
g2696pR	CCC **AGATCT**CATTGTACTAGTTATATTTTAAATTATTTTAGTAATGTTAC	
g2380pF	GTGTCAGACATTTTATGAATATATATAGTTA **TCTAGA**AAG	*psp3*
g2380pR	CC **AGATCT**TGGCTTCATTTATTTATGTATATGTG	
Gal17F	TATGGTAAAACTTGAATTGATCCTCTAG	
Gal17R	CATCCTGCAGTAAGCTTGGTACCGAGATC	

Transformants were initially selected at 50 μg/ml G418 (G4181, Formedium) on 1/3 ^rd^ SM agar with G418 resistant *Escherichia coli* ( Narita *et al.*, 2020), which was raised to 100 μg/ml G418 for *psp1-lacZ* and *psp2-lacZ* transformed cells and to 200 μg G418/ml for *psp3-lacZ*, which was expressed relatively weakly. The generated plasmids are deposited in the *Dictyostelium* Stock Center http://dictybase.org/StockCenter/StockCenter.html.

### β-galactosidase histochemistry

Cells transformed with *lacZ* constructs were washed free from bacteria and 20 μl aliquots of 10 ^8^ cells/ml were plated on 2×2 cm squares of nitrocellulose filters (GE10600002, Merck) supported by NN agar. Filters with developing structures were transferred to squares of Whatman 3MM paper (WHA3030931, Merck) soaked in 0.5% glutaraldehyde (354400, Sigma-Aldrich) in Z buffer (60 mM Na _2_HPO _4_, 40 mM NaH _2_PO _4_ (71469, Sigma-Aldrich), 10 mM KCl (P3911, Sigma-Aldrich) and 1 mM MgSO _4_, pH 7.0) and incubated for 5 min. Structures were next fully submersed in 0.25% glutaraldehyde and 1% Tween-20 (P2287, Sigma-Aldrich) in Z buffer for 10 min and then washed and stained with X-gal (XGAL001, Formedium) as described previously ( Dingermann *et al.*, 1989).

### Prespore gene induction and β-galactosidase assay

For time courses, *Pvio* cells transformed with *psp1-LacZ* or *psp2-LacZ* were developed on nitrocellulose filters as above, harvested from filters by mixing on a Vortex Genie 2 (Z258423, Sigma-Aldrich) in 0.5 ml 1x Z-buffer, frozen at -20°C and lysed by freeze-thawing three times, with thawing under vigorous shaking. β-galactosidase activity was assayed in 100 μl lysate and protein in 20 μl lysate in triplicate.

For gene induction cells were starved for 4 h on NN agar, aggregates were gently dissociated and cells resuspended to 2×10 ^6^ cells/ml. 50 μl of cell suspension was incubated in triplicate with 50 μl of variables or water in microtiter plates for 6 h at 22°C, while shaking on an IKA MTS4 microplate shaker (0003208002 IKA, Oxford, UK). Cells were frozen at -20°C and lysed as above.

To assay β-galactosidase activity, lysed cells were supplemented with 10 μl 40 mM chlorophenolred-β-D-galactopyranoside (CPRG)(59767 Sigma-Aldrich) and for the gene induction experiments with 30 μl 2.5x Z-buffer (1% mercapto-ethanol (M3148, Sigma-Aldrich) in 150 mM Na _2_HPO _4_, 100 mM NaH _2_PO _4_, 25 mM KCl, 7.5 mM MgSO _4_, pH 7.0). The optical density at 574 nm was measured at regular intervals using a Multiskan SkyHigh Microplate Spectrophotometer (A51119500C, Thermo Fisher Scientific). The β-galactosidase activity (ΔOD _574_/min) was calculated from one or more intervals where OD _574_ increased linearly and standardized on the protein content of the lysates as assayed with Bradford reagent (5000205 Bio-rad).

## Results

### Identification of *Pvio* prespore genes

*D. discoideum* prespore genes were originally identified as genes that were highly enriched in the prespore fraction of dissociated slugs, separated on density gradients ( Mehdy *et al.*, 1983; Ratner & Borth, 1983). Many of these genes were found to encode spore coat proteins, which accumulate as an inner lining of Golgi-derived prespore vesicles in the posterior of early slugs ( Fosnaugh & Loomis, 1989). In the final phase of fruiting body formation, the vesicles fuse with the plasma membrane and their inner lining forms the first layer of the spore wall. Another prespore gene, *pspA*, encodes a cell surface protein, recognized by monoclonal antibody MUD1 ( Early *et al.*, 1988). The expression of all these genes starts shortly after aggregation and is induced and maintained by micromolar concentrations of cAMP ( Mehdy & Firtel, 1985; Schaap & Van Driel, 1985). The genes encoding the vesicle-associated proteins also require intracellular cAMP acting on PKA for expression ( Hopper *et al.*, 1993).

**Figure 1.**
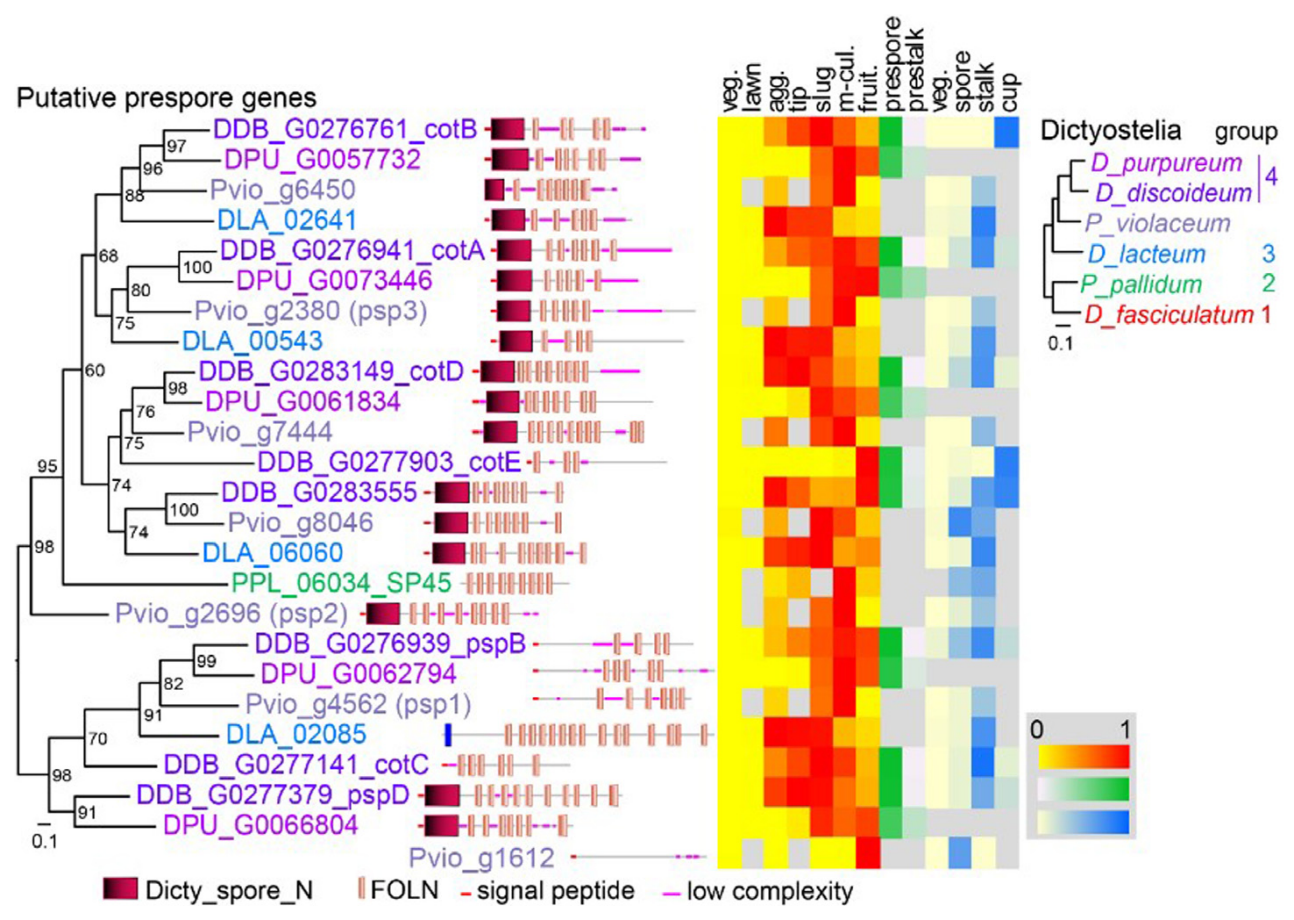
Search for *Pvio* prespore genes. The *Pvio* ( Narita *et al.*, 2020), *D. lacteum* ( Glöckner *et al.*, 2016) and *D.purpureum* ( Sucgang *et al.*, 2011) proteomes were queried for relatives of 7 well-documented *D. discoideum* and one *P. pallidum* prespore genes using BLASTp. The top hits were aligned using ClustalOmega ( Sievers & Higgins, 2014) and a phylogenetic tree was inferred with IQtree ( Nguyen *et al.*, 2015). The tree is annotated with the functional domains of the proteins, as analysed by SMART ( Schultz *et al.*, 1998), and with relative expression levels of the genes during the *Ddis* developmental cycle and in its purified cell types, as retrieved from published RNAseq experiments ( Glöckner *et al.*, 2016; Kawabe *et al.*, 2023; Kin *et al.*, 2018; Narita *et al.*, 2020; Parikh *et al.*, 2010). A multigene phylogeny of taxon-group representative Dictyostelia ( Schilde *et al.*, 2019) is shown in top right corner.

The spore coat proteins are members of a large family that share FOLN (Follistatin-N-terminal domain-like, EGF-like) domains and often Dicty_spore_N domains. The family shows gene gain and loss in the different dictyostelid taxon groups, precluding unequivocal assessment of orthology. While many *Ddis* members have known functions in spore coat assembly ( Srinivasan *et al.*, 1999; West, 2003), functions in the stalk or cyst wall for others cannot be excluded. To identify *Pvio* prespore genes, I therefore sought for the closest relatives of the well-characterized *Ddis* spore coat genes *cotA*, *cotB*, *cotC*, *cotD*, *cotE*, *pspA*, *pspB* and *pspD* ( Fosnaugh & Loomis, 1989; Grant *et al.*, 1985; Metcalf *et al.*, 2007; Smith *et al.*, 1989; Takemoto *et al.*, 1990; Yoder *et al.*, 1994) and *SP45,* a prespore gene of the group 2 species *Ppal* ( Gregg & Cox, 2000). To assist subdivision in orthologous groups, a second group 4 species, *D. purpureum* ( *Dpur*), and the group 3 species *D. lacteum* ( *Dlac*) were also included in the search. A phylogeny was inferred from the closest BLASTp hits to the 7 *Ddis* genes, which was annotated with protein functional domains and developmental and cell-type specific expression profiles of the genes ( Figure 1). *PspA* was not detected in the *Pvio* genome. The relatives of *cotB*, *cotA*, *cotD* and *pspB* separate into clades with single members in each investigated species. Across species, these genes and most of the others increase expression in aggregates, but down-regulate expression in mature fruiting bodies. While in *Ddis* slugs, their expression is strongly prespore-enriched, later in mature fruiting bodies, expression is higher in cup and stalk cells than in spores. Note that the developmental- and cell-type specificity profiles are obtained from separate RNAseq experiments, which means that expression data between profiles cannot be compared in an absolute sense and expression in all mature cell types may be relatively low. *cotE* is expressed late in development and is directly transported to the spore coat ( Metcalf *et al.*, 2007). A previously identified *Pvio* spore gene *Pvio_g1612* ( Narita *et al.*, 2020) belongs to a similar class of late spore genes. From the six detected *Pvio* prespore gene candidates, I selected three genes *Pvio_*g4562 ( *psp1*), *Pvio_*g2696 ( *psp2*) and *Pvio_*g2380 ( *psp3*) with relatively high expression levels and for which the full 5’intergenic sequence was present in the (draft) genome. 

### Expression pattern of putative *Pvio* prespore genes

**Figure 2.**
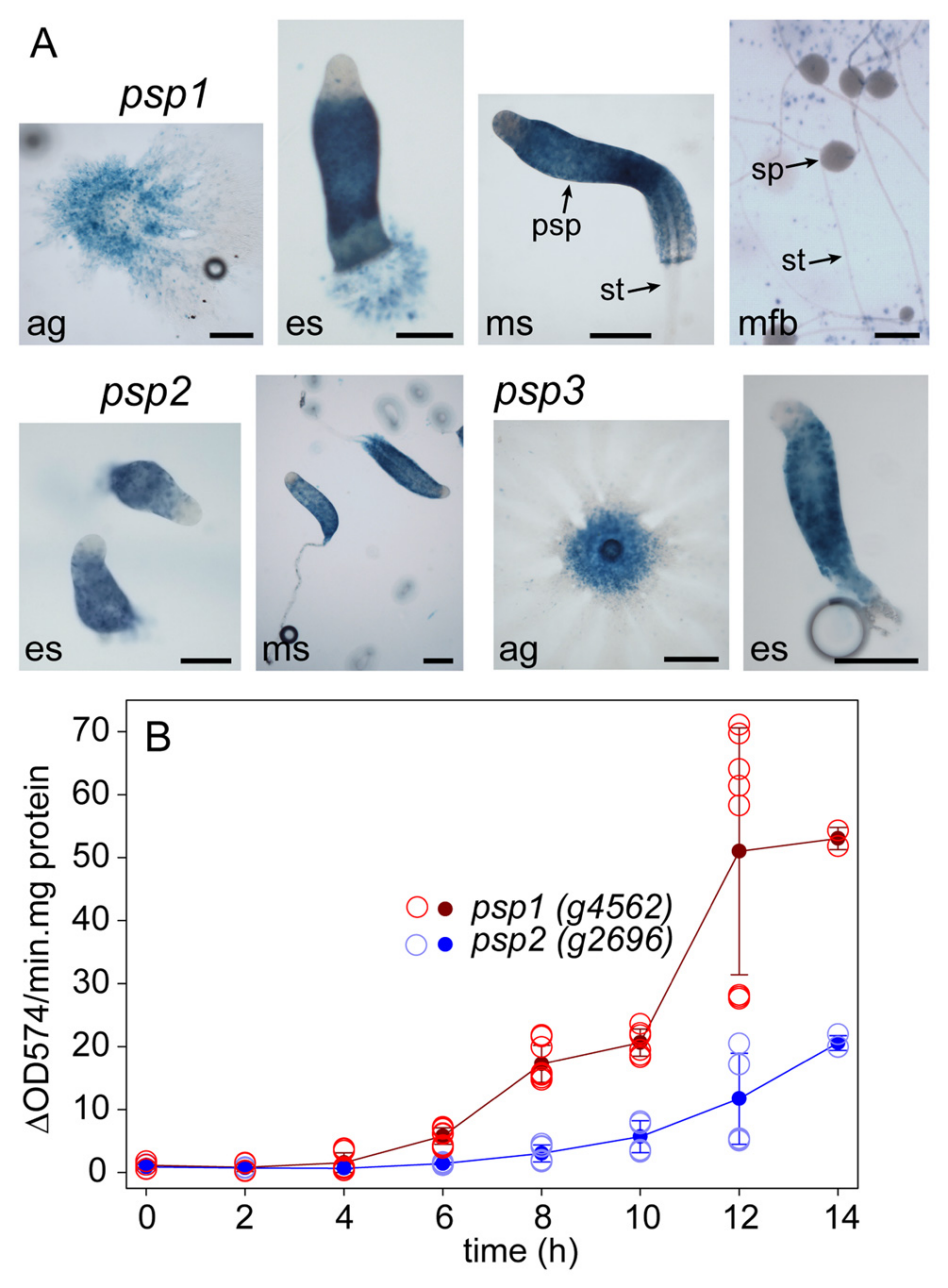
Pattern and timing of *Pvio* prespore gene expression. **A**. *Pattern. Pvio* cells transformed with *lacZ* fusions with the 5’intergenic regions of the *Pvio psp1* ( *g4562*), *psp2* ( *g2696*) and *psp3* ( *g2380*) genes were developed on nitrocellulose filters supported by NN agar. Developing structures were fixed and stained with X-gal from 4 h and imaged under transmitted light. Bars: 200 μm. ag: aggregates, es and ms: early and mid sorogens, mfb: mature fruiting bodies, psp, sp and st: prespore, spore and stalk cells. **B**. *Developmental timing.* 4x10 ^6^ cells developed on 2x2 cm ^2^ filters were harvested and frozen at the indicated time periods of starvation, and then lysed and assayed spectrophotometrically for β-galactosidase activity as described in Methods. Two time courses with triplicate filters were examined for the *psp1-LacZ* cells and one time course for the *psp2-LacZ* cells. The open circles represent the activity standardized on protein of the individual filters and the closed circles and error bars the mean and SD of all measurements. The underlying data are archived at https://osf.io/bawv6/ PvioPresporeGeneInduction.xlsx, sheet Fig.2BData. ( Schaap, 2024).

The 5’intergenic regions of the three candidate prespore genes were fused to the *lacZ* reporter gene and *Pvio* cells, transformed with either construct, were developed on non-nutrient agar and stained with X-gal ( Figure 2A). The promoter activity patterns of the three genes were very similar. β-galactosidase activity was first observed inside newly formed aggregates with little to no activity in the inflowing streams and then partitioned to the rear 4/5 ^th^ of the emerging sorogen (es). The tip region, which was initially free of β-galactosidase activity, became proportionally smaller as the sorogen matured and the newly formed stalks contained few or no stained cells. While in freshly formed fruiting bodies the spore heads were deeply stained, β-galactosidase activity disappeared as the spores matured further.

Overall the expression pattern of the *Pvio* prespore genes was similar to that of the *Ddis* prespore genes *pspA* and *cotA,B* and *C,* except the anterior prespore free region remains considerably larger in *Ddis* throughout development. Compared to *Ddis* sorogens, the signature prespore vesicles are also present much closer to the tip in *Pvio* sorogens, where they undergo degradation as the cells are differentiating into stalk cells ( Schaap *et al.*, 1985).

Genes *psp1*, *psp2* and *psp3* are therefore reliable markers of prespore differentiation.

To estimate the developmental stage when cells were competent for gene induction by signal molecules, *psp1-lacZ* and *psp2-lacZ* cells, developed on filters, were also assayed for β-galactosidase activity by a quantitative spectrophotometric assay. Figure 2B showed that activity started to increase at 4 h of starvation (when cells had formed streaming aggregates) up to the last time point at 14 h (when cells were in the mid-sorogen stage). *Psp1-lacZ* cells showed about 2.5 fold higher activity as *psp2-lacZ* cells.

### Effects of *Ddis* and *Pvio* signal molecules on *Pvio* prespore gene expression

Prespore gene expression in *Ddis* is induced by cAMP acting on cARs and further promoted by GABA (gamma-aminobutyric acid), an unknown steroid and the peptides SDF1 and SDF2. The latter four signals eventually activate PKA, which is also directly activatable by the membrane-permeant cAMP analogue 8Br-cAMP ( Anjard *et al.*, 1998; Anjard & Loomis, 2006; Anjard & Loomis, 2008; Anjard *et al.*, 2009; Wang *et al.*, 1999). c-di-GMP activates PKA in prestalk cells to induce stalk cell maturation ( Chen & Schaap, 2012; Chen *et al.*, 2017), while DIF-1 induces basal disc differentiation in *Ddis* ( Saito *et al.*, 2008). cAMP and glorin are the chemoattractants that mediate *Ddis* and *Pvio* aggregation respectively, while folate mediates attraction to the bacterial food source ( Konijn *et al.*, 1967; Pan *et al.*, 1972; Shimomura *et al.*, 1982). cGMP is a second messenger mediating chemotaxis ( Mato *et al.*, 1977). Except for SDF1 and SDF2 which are not commercially available, I compared the effect of all these compounds on the induction of *Pvio psp1-lacZ* and *psp2-lacZ* expression in 4 h starved cells shaken in buffer for 6 h with cells developing on solid substratum for the same period. The compounds were added in the concentration range where they are active in *Ddis*. These are 10 ^-5^ to 10 ^-3^ M for cAMP ( Schaap & Van Driel, 1985), >10 ^-10^ M for DIF-1 ( Masento *et al.*, 1988), 10 ^-7^ to 10 ^-5^ M for c-di-GMP ( Chen & Schaap, 2012), 10 ^-9^ to 10 ^-4^ M for GABA ( Anjard & Loomis, 2006), 10 to 15 mM for 8Br-cAMP ( Yamada *et al.*, 2018). Glorin, folate and cGMP were used in the range where cAMP induces prespore gene expression.

**Figure 3.**
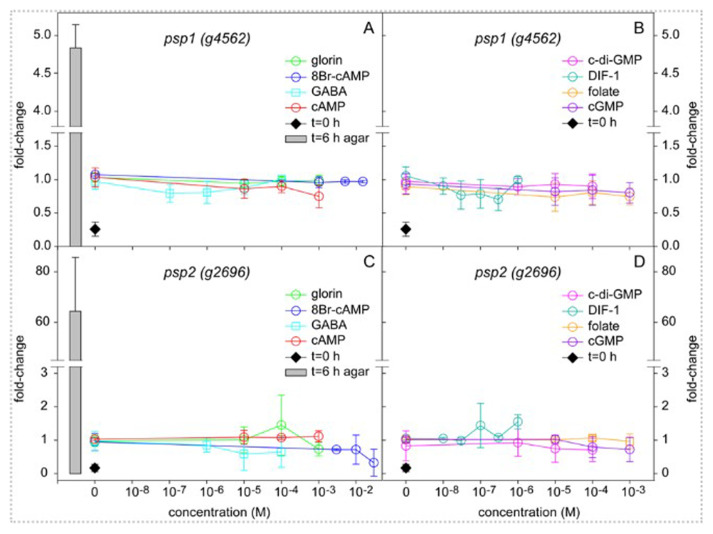
Induction of prespore gene expression by signal molecules. *Pvio psp1-lacZ* and *psp2-lacZ* cells were starved for 4 h on NN agar, harvested and incubated at 10 ^6 ^cells/ml in PB for 6 h with the indicated concentrations of signal molecules or developed on as 10 ^6^ cells per 1 cm ^2^ filter on NN agar. At 0 and 6 h of incubation cells were frozen, lysed and assayed for β-galactosidase activity and protein content. The results are expressed as fold-change of the ΔOD _574_/min.mg protein values over the averaged control (water) values for all concentration series at t=6 h. Data represent means and SD of 2 or 3 independent experiments performed in triplicate for each variable. The underlying data are archived at https://osf.io/bawv6/ PvioPresporeGeneInduction.xlsx, sheet Fig.3Data ( Schaap, 2024).

Figure 3 shows that without added compounds *psp1-lacZ* activity increased about 4-fold over 6 h incubation, while *psp2-lacZ* activity increased about 6-fold (compare black diamonds to coloured circles at 0 M). None of the added compounds had any significant effect on this increase over their entire concentration range. Note that the control (water) values fluctuate a bit around 1 for the individual concentration series, which likely reflects accumulated pipetting errors, since this value was set at 1 for the averaged control values of all series. The fluctuations from 1 when variables were added is mostly not greater than that at the control, indicating that they represent the pipetting error. On the other hand, the cells developed for 6 h on solid substratum (to mid-sorogens) showed 5 to 60-fold higher activity for *psp1* and *psp2*, respectively, than those incubated in dilute suspensions.

Evidently, the cells received some signal during normal development, that is different from any of the signals that were added during incubation in suspension, although a small amount of an endogenous inducer was also produced by the suspended cells.

## Discussion

Three marker genes for prespore differentiation were identified in *Pvio*. Their promoters were fused to *LacZ* and *a* convenient bioassay for detection of prespore-inducing signals was developed from *Pvio* cells expressing these constructs. Unlike *Ddis* in group 4 ( Schaap & Van Driel, 1985) and *Ppal* in group 2 ( Kawabe *et al.*, 2009) who use secreted cAMP acting on cARs to induce prespore genes, *Pvio,* a sister species to group 4 did not require its supposedly single cAR for (pre)spore differentiation ( Kawabe & Schaap, 2023). Using the novel constructs, it was evident that *Pvio* prespore genes are not inducible by cAMP ( Figure 3A,C), which indicates that normal development of the *Pvio cAR* knock-out was not due to an undiscovered cAR taking over its function.

The *Pvio* chemoattractant glorin could also not induce expression of prespore genes and neither did folate, a chemoattractant used for food seeking in group 4 ( Pan *et al.*, 1972) and for aggregation in the group 3 species *D. minutum* ( De Wit & Konijn, 1983). Dissimilar to *Ddis*, the *Pvio* prespore-inducing signal is unlikely to be a chemoattractant. cGMP, an intermediate of cAMP- and folate-induced chemotaxis ( Mato *et al.*, 1977) had also no effect on prespore gene induction. Competent *Pvio* cells showed a low level of prespore gene induction in suspension, but this endogenous induction was neither stimulated nor inhibited by the *Ddis* signals c-di-GMP and DIF-1 ( Figure 3B,D) which induce stalk and basal disc expression, respectively ( Chen & Schaap, 2012; Saito *et al.*, 2008), and in case of DIF-1 inhibit prespore differentiation ( Wang *et al.*, 1987).

Many signals act late in *Ddis* development to regulate the timely maturation of spore and stalk cells by controlling the levels of intracellular cAMP and thereby the activation of PKA. These signals such as GABA, SDF1 and SDF2 and an unidentified steroid act in synergy with cAR activation by extracellular cAMP to induce maximal expression of prespore genes and exocytosis of prespore vesicles, which are lined with the first layer of the spore coat. The effects of these late signals can be mimicked by the membrane-permeant PKA agonist 8Br-cAMP ( Anjard *et al.*, 1998; Anjard & Loomis, 2006; Anjard & Loomis, 2008; Anjard *et al.*, 2009; Wang *et al.*, 1999). A secreted glycoprotein, PsiA, promotes prespore differentiation in the presence of cAMP in an unknown manner ( Kawata *et al.*, 2004). *psiA* is member of a clade of 15 PA14 genes, which are amplified only in group 4, and it therefore has no ortholog in *Pvio*.

Neither GABA nor up to 30 mM of 8Br-cAMP stimulated *Pvio* prespore gene expression. *Pvio* cells can take up to 8Br-cAMP, since earlier experiments showed that 10 mM 8Br-cAMP could restore aggregation and spore differentiation in *Pvio* lacking the two adenylate cyclases *acaA* and *acrA*. In *Ddis*, *AcrA* has a specific role in synthesizing cAMP for PKA activation in spore maturation and the late functions of *AcrA* and PKA were apparently conserved in *Pvio* ( Kawabe & Schaap, 2023). Only the early role of secreted cAMP in prespore induction is not conserved and the question arises what the missing signal might be.

Dictyostelia contain many species- or group-specific genes involved secondary metabolism such as polyketide synthases and terpene synthases ( Chen *et al.*, 2018; Heidel *et al.*, 2011; Zucko *et al.*, 2007). Several secondary metabolites were previously identified that act on cell differentiation, such as DIF-1 ( Morris *et al.*, 1987), MPBD ( Narita *et al.*, 2011) and dictyodene D ( Günther *et al.*, 2022). In addition, dictyostelid genomes contain large and highly variable families of cell surface or secreted glycoproteins, such as the IPT/TIG and PA40 domain families with known members like tgrB1/C1 and psiA being involved in developmental transitions ( Hirose *et al.*, 2015; Kawata *et al.*, 2004). In the assay used here cells still clumped together during incubation, making it unlikely that direct cell-cell contact was the missing signal. While it will be a daunting task to identify any candidate as the *Pvio* prespore inducer, the availibitily of a sensitive bioassay based on the *psp1_LacZ* transformed *Pvio* cells is a step towards its completion.

The loss of secreted cAMP function in *Pvio* multicellular development does not only concern prespore induction but also the regulation of multicellular morphogenesis, which is in groups 4 ( Singer *et al.*, 2019), 3 ( Alvarez-Curto *et al.*, 2005; Schaap *et al.*, 1984) and 2 ( Kawabe *et al.*, 2009; Kawabe *et al.*, 2012) mediated by cAMP as chemoattractant. With the associated proteins involved in the timely synthesis and processing of the novel signal(s), this is from an evolutionary perspective an immense lineage-specific innovation in a pathway essential for species survival. It emphasizes that evolution does not always involve small stepwise improvements, but can proceed in remarkably large spurts.

## Ethics and consent

Ethical approval and consent were not required.

## Data Availability

OSF: Prespore gene induction in Polysphondylium violaceum. DOI 10.17605/OSF.IO/BAWV6 This project contains the following underlying data: A spreadsheet entitled PvioPresporeGenInduction.xlsx This project is under license by *CC0 1.0 Universal* The generated plasmids are deposited in the *Dictyostelium* Stock Center http://dictybase.org/StockCenter/StockCenter.html.
