# Supplementary material for: NOVEL INVENTION OF SPORE INDUCTION IN A SISTER SPECIES TO GROUP 4 DICTYOSTELIA
Source: Open Res Eur. 2024 Dec 5;4:239. Originally published 2024 Oct 29. [Version 2] doi: 10.12688/openreseurope.18365.2 (PMC11574339; doi:10.12688/openreseurope.18365.2)
Supplement: Peer review history

# Reviewer report: Reviewer response for version 2

**DOI:** 10.21956/openreseurope.20582.r48164

**Published:** 2024-12-14

**Authors:** Yusuke V. Morimoto

**Affiliations:** 1 Kyushu Institute of Technology, Fukuoka, Japan

The author has made sufficient additions and corrections and this manuscript should be index as a final version.

Is the study design appropriate and does the work have academic merit?

Yes

Is the work clearly and accurately presented and does it cite the current literature?

Partly

If applicable, is the statistical analysis and its interpretation appropriate?

Yes

Are all the source data underlying the results available to ensure full reproducibility?

Yes

Are the conclusions drawn adequately supported by the results?

Partly

Are sufficient details of methods and analysis provided to allow replication by others?

Yes

Reviewer Expertise:

Molecular genetics, Cell biology, Biophysics

I confirm that I have read this submission and believe that I have an appropriate level of expertise to confirm that it is of an acceptable scientific standard.

# Reviewer report: Reviewer response for version 1

**DOI:** 10.21956/openreseurope.19845.r45744

**Published:** 2024-11-18

**Authors:** Shweta Saran

**Affiliations:** 1 Jawaharlal Nehru University, New Delhi, Delhi, India

The article entitled “Novel invention of spore induction in a sister species to group 4 Dictyostelia” by Pauline Schaap is accepted.

 The main findings in this article are the following:

- She identified the *Polyspondylium violaceum (Pvio)* genes g4562 (psp1), g2696 (psp2) and g2380 (psp3) which were homologs of *Dictyostelium discoideum* spore coat genes and studied their expression patterns.
- cAMP had no effect on prespore gene induction and neither had the *Pvio* chemoattractant glorin nor the *Dictyostelium discoideum* chemoattractants and differentiation inducers folate, c-di-GMP, DIF-1, GABA, cGMP and 8Br-cAMP.

Thus, she concluded that *Pvio* lineage uniquely evolved a novel genetic network for synthesis, detection and processing of the unknown signal in a timely manner that triggers its main survival strategy.

 The manuscript is well written, experiments are neatly designed and figures are clear. The discussion is very well written. There are earlier paper (Hanna *et al*., Expt Cell Res, 1979, 122:265-271; Shimomura *et al*., 1982, PNAS 79: 7376-7379) where they have talked about morphogenesis and stalk and spore formation that is independent of cAMP (the known chemoattractant in *D. discoideum*, belonging to the same group IV). In her earlier paper, (Kawabe and Schaap, Biol Open, 2023, 12), they have shown that in *Pvio* secreted cAMP was not required for development but intracellular cAMP was required for the initiation of development and spore maturation. This study was an outcome of many deletion mutants generated that were components of cAMP signalling pathway. Here an elegant method was employed to study the different spore genes.

  I was just wondering if any metabolites could be involved?

Is the study design appropriate and does the work have academic merit?

Yes

Is the work clearly and accurately presented and does it cite the current literature?

Yes

If applicable, is the statistical analysis and its interpretation appropriate?

Yes

Are all the source data underlying the results available to ensure full reproducibility?

Yes

Are the conclusions drawn adequately supported by the results?

Yes

Are sufficient details of methods and analysis provided to allow replication by others?

Yes

Reviewer Expertise:

cell and developmental biology working with Dictyostelium

I confirm that I have read this submission and believe that I have an appropriate level of expertise to confirm that it is of an acceptable scientific standard.

## References

[1] : Development of the dictyostelid Polysphondylium violaceum does not require secreted cAMP. *Biol Open* .2023;12(2) : 10.1242/bio.059728 10.1242/bio.059728 36688866 PMC9922732

# Response

**Published:** 2024-11-23

**Authors:** Pauline Schaap

**Affiliations:** University of Dundee, UK

I thank reviewer 3 for her concise description and positive opinion of the manuscript. With regard to her question whether any metabolites could be involved, I assume that she meant metabolites of the compounds that were tested. This is possible, but one then would assume at least some effect of the added variables as they would likely be metabolized during the 6 h incubation period.

# Reviewer report: Reviewer response for version 1

**DOI:** 10.21956/openreseurope.19845.r45746

**Published:** 2024-11-14

**Authors:** Yusuke V. Morimoto

**Affiliations:** 1 Kyushu Institute of Technology, Fukuoka, Japan

This study suggests the existence of an unknown signaling pathway in cellular slime molds, a finding that is of great evolutionary interest both in the study of cellular slime molds and in the study of eukaryotic signal transduction more broadly. The draft is carefully verified in comparison with previous findings. However, some of the descriptions are inadequate and need to be revised.

 1. In the results of the *lacZ* reporter, data corresponding to the description are not adequately presented.“β-galactosidase activity was first observed in the centre of streaming aggregates”

 This is shown by the photo of the ag of psp3 in Fig 2A? However, the same activity is not seen in the ag of psp1.

 “the newly formed stalks contained few or no stained cells.”

 Do you have any pictures showing this?

 “While in freshly formed fruiting bodies the spore heads were deeply stained, β-galactosidase activity

 disappeared as the spores matured further.”

 Arrows are needed to indicate where to look in Fig 2A. Need descriptions such as which parts are the spores.

 2. Fig2B and Fig3 do not show about *psp3*. Can you explain why only *psp1* and *psp2* were used?

 3. Since it is mentioned that “The compounds were added in the concentration range where they are active in *Ddis*.”, references are needed for each active concentration of each compounds.

 4. I could not find the numbers in the following text from the Figure 3. “Figure 3 shows that without added compounds psp1-lacZ activity increased about 4-fold over 6 h incubation, while psp2-lacZ activity increased about 6-fold.”

 Please provide a detailed explanation or modify the figure to make them easier to read.

 And, it should be shown that there is no change in the water-only control.

 5. The activity values for *psp1* and *psp2* in Fig3 are reversed from those in Fig2. A description of what causes this difference is needed.

 6. Page6, Line13: “stalk cels” should be “stalk cells”.

 7. Page7, Line2 “psp1-lacZ” and “psp1-lacZ” should be in italic.

Is the study design appropriate and does the work have academic merit?

Yes

Is the work clearly and accurately presented and does it cite the current literature?

Partly

If applicable, is the statistical analysis and its interpretation appropriate?

Yes

Are all the source data underlying the results available to ensure full reproducibility?

Yes

Are the conclusions drawn adequately supported by the results?

Partly

Are sufficient details of methods and analysis provided to allow replication by others?

Yes

Reviewer Expertise:

Molecular genetics, Cell biology, Biophysics

I confirm that I have read this submission and believe that I have an appropriate level of expertise to confirm that it is of an acceptable scientific standard, however I have significant reservations, as outlined above.

# Response

**Published:** 2024-11-23

**Authors:** Pauline Schaap

**Affiliations:** University of Dundee, UK

*I am grateful to the reviewer for his in-depth review. Revisions were made to the manuscript to address his comments as outlined below:*

 1. In the results of the lacZ reporter, data corresponding to the description are not adequately presented.“β-galactosidase activity was first observed in the centre of streaming aggregates” This is shown by the photo of the ag of psp3 in Fig 2A? However, the same activity is not seen in the ag of psp1.

***Au:** I agree that the description allowed multiple interpretations. I meant that it was observed in the actual aggregate and not the inflowing streams. The sentence was altered into:”* *β-galactosidase activity was first observed inside newly formed aggregates with little to no activity in the inflowing streams”.*

 “the newly formed stalks contained few or no stained cells.” Do you have any pictures showing this? *Au: this was evident in figure 2A right top panels, but is due to its low resolution not clear in the published PDF version. I have replaced the third top panel with a figure at higher magnification.  * “While in freshly formed fruiting bodies the spore heads were deeply stained, β-galactosidase activity disappeared as the spores matured further.” Arrows are needed to indicate where to look in Fig 2A. Need descriptions such as which parts are the spores.

***Au: **Arrows and descriptions are added to figure 2A*

 2. Fig2B and Fig3 do not show about psp3. Can you explain why only psp1 and psp2 were used?

***Au**: The psp3-LacZ plasmid took much longer to construct than the other two and was only finished after the induction experiments were mostly done. The psp3 promoter activity was also very weak and we therefore did not consider it necessary to repeat all experiments again with this promoter.*

 3. Since it is mentioned that “The compounds were added in the concentration range where they are active in Ddis.”, references are needed for each active concentration of each compounds.

***Au**: This sentence is now followed up by a referenced listing of active concentration ranges* 

 4. I could not find the numbers in the following text from the Figure 3. “Figure 3 shows that without added compounds psp1-lacZ activity increased about 4-fold over 6 h incubation, while psp2-lacZ activity increased about 6-fold.” Please provide a detailed explanation or modify the figure to make them easier to read.

***Au**: a similar comment (#2) was also made by reviewer 1. I added the line “(compare black diamonds to coloured circles at 0 M)” to the text to clarify this. The black diamonds are controls taken at t=0 h and the coloured circles at this position are water controls taken at t=6h.* *Note that the underlying experimental data can be accessed in a spreadsheet using hyperlink* *https://osf.io/bawv6/* *, as indicated in the legend to figure 3* And, it should be shown that there is no change in the water-only control. *Au: the change between 0 and 6 h at 0 M are the roughly 4 and 6-fold changes in basal activity*

 5. The activity values for psp1 and psp2 in Fig3 are reversed from those in Fig2. A description of what causes this difference is needed.

***Au**: This seems particularly the case for cells developed on agar. In figure 2B the β-galactosidase activity is expressed as an absolute value (* *ΔOD574/min.mg protein), while in  figure 3 the activity is expressed as fold-change over the water control value to highlight the extent of gene induction (or lack thereof) by the different variables.  The high value for psp2 agar control simply reflects that compared to psp1 its water control value (in the denominator) is very low, thus producing a high fold-change.*

 6. Page6, Line13: “stalk cels” should be “stalk cells”.

***Au:** corrected*

 7. Page7, Line2 “psp1-lacZ” and “psp1-lacZ” should be in italic

***Au:** corrected*

# Reviewer report: Reviewer response for version 1

**DOI:** 10.21956/openreseurope.19845.r45740

**Published:** 2024-11-13

**Authors:** Tamao Saito

**Affiliations:** 1 Sophia University, Tokyo, Japan

Molecular phylogenetic analysis by the author has divided cellular slime mould into four groups. The most well-studied D. discoideum (Ddis) belongs to the most evolved Group 4 (G4).

 Previous studies have suggested that there is a significant evolutionary gap between G4 and G3. Therefore, the analysis of morphological formation using P. violaceum (Pvio), a sister clade of G4, is important in tracing the path by which G4 evolved from G3.

 Finding differentiation markers is therefore a fundamental and essential step. The author identified spore markers based on meticulous validation and showed the pattern formation and the timing of marker gene expression. In addition, bio-assay have been established that use these markers to find signaling molecules. The bioassay method is particularly useful for Pvio, as it is conceivable that some signaling molecules are the same as those of Ddis but work through different pathways.

 In this study, a major step in the evolution between G4 and G3 was demonstrated.

 I only have a few suggestions and question. None of these are major.

 Page 6 fifth line from bottom of left column. Shouldn't “we” be changed to “I”?

 Page 7 last sentence; In Figure 3, it was not clear where the activity of each gene was shown to increase by 4- to 6-fold without the added compounds.

 I would like to comment on the signaling molecules involved in the expression of the prespore genes. Among the secondary metabolites listed in the Discussion, MPBD and Dictyoquinone have been shown to be involved in cAMP signaling in Ddis and also in spore maturation. In this context, it would be appropriate to investigate their effects in this study.

Is the study design appropriate and does the work have academic merit?

Yes

Is the work clearly and accurately presented and does it cite the current literature?

Yes

If applicable, is the statistical analysis and its interpretation appropriate?

Yes

Are all the source data underlying the results available to ensure full reproducibility?

Yes

Are the conclusions drawn adequately supported by the results?

Yes

Are sufficient details of methods and analysis provided to allow replication by others?

Yes

Reviewer Expertise:

Biochemistry,  Chemical ecology

I confirm that I have read this submission and believe that I have an appropriate level of expertise to confirm that it is of an acceptable scientific standard.

# Response

**Published:** 2024-11-23

**Authors:** Pauline Schaap

**Affiliations:** University of Dundee, UK

I thank the reviewer for her insightful review. I made minor revisions to the manuscript and respond to her numbered comments below:  

1. Page 6 fifth line from bottom of left column. Shouldn't “we” be changed to “I”?

Au: This and three more instances of “we” were replaced by “I”  

1. Page 7 last sentence; In Figure 3, it was not clear where the activity of each gene was shown to increase by 4- to 6-fold without the added compounds.

Au: This is evident by comparing the black diamonds (t=0 h) with the coloured circles (t=6 h) at 0 M of additives. I have added a note in the text to clarify this.  

1. I would like to comment on the signaling molecules involved in the expression of the prespore genes. Among the secondary metabolites listed in the Discussion, MPBD and Dictyoquinone have been shown to be involved in cAMP signaling in Ddis and also in spore maturation. In this context,  it would be appropriate to investigate their effects in this study.

Au: MPBD was shown to promote spore maturation by indirectly activating PKA. We here tested other compounds (GABA and 8Br-cAMP) that promote spore maturation by activating PKA. They had no effect on prespore gene induction, so we did not consider it necessary to test more compounds in this category. Dictyoquinone is to our knowledge not commercially available.
